# 3D‐Printed Artificial Cilia Arrays: A Versatile Tool for Customizable Mechanosensing

**DOI:** 10.1002/advs.202303164

**Published:** 2023-07-23

**Authors:** Phillip Glass, Andy Shar, Charles Pemberton, Ethan Nguyen, Sung Hyun Park, Daeha Joung

**Affiliations:** ^1^ Department of Physics Virginia Commonwealth University Richmond VA 23284 USA; ^2^ Sustainable Technology and Wellness R&D Group Korea Institute of Industrial Technology (KITECH) Jeju‐si Jeju‐do 63243 Republic of Korea; ^3^ Massey Cancer Center Virginia Commonwealth University Richmond VA 23298 USA; ^4^ Institute for Sustainable Energy and Environment Virginia Commonwealth University Richmond VA 23284 USA

**Keywords:** 3D printing, cilia arrays, graphene composites, mechanosensors, tactile sensors

## Abstract

Bio‐inspired cilium‐based mechanosensors offer a high level of responsiveness, making them suitable for a wide range of industrial, environmental, and biomedical applications. Despite great promise, the development of sensors with multifunctionality, scalability, customizability, and sensing linearity presents challenges due to the complex sensing mechanisms and fabrication methods involved. To this end, high‐aspect‐ratio polycaprolactone/graphene cilia structures with high conductivity, and facile fabrication are employed to address these challenges. For these 3D‐printed structures, an “inter‐cilium contact” sensing mechanism that enables the sensor to function akin to an on‐off switch, significantly enhancing sensitivity and reducing ambiguity in detection, is proposed. The cilia structures exhibit high levels of customizability, including thickness, height, spacing, and arrangement, while maintaining mechanical robustness. The simplicity of the sensor design enables highly sensitive detection in diverse applications, encompassing airflow and water flow monitoring, braille detection, and debris recognition. Overall, the unique conductive cilia‐based sensing mechanism that is proposed brings several advantages, advancing the development of multi‐sensing capabilities and flexible electronic skin applications in smart robotics and human prosthetics.

## Graphical abstract

A new strategy is proposed to fabricate and detect artificial cilia sensors through 3D printing. Polycaprolactone/graphene nanocomposites can be used to create multifunctional mechanosensors to detect mechanical disruptions through contact‐initiated current flow. This cost‐efficient fabrication technique allows for the construction of customizable sensors, and can be used in sensing brushes, artificial eyelashes, air and water flow detection, and beyond.

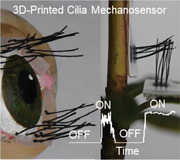

## 1 Introduction

Mechanosensors are essential for robotic detection of environmental stimuli in electronic skin (e‐skin) applications and minimally invasive surgery, allowing robots to sense changes in pressure, airflow, temperature, humidity, as well as mapping surface topology and effecting rapid response to dust or debris.^[^ ^1^, ^2^, ^3^, ^4^, ^5^, ^6^, ^7^, ^8^ ^]^ Within the field of mechanosensing, artificial cilia sensors have emerged as a new way of sensing and responding to changes in the environment, with potential applications ranging from environmental sensing to biomedical diagnostics and therapeutics.^[^ ^9^, ^10^, ^11^, ^12^, ^13^ ^]^ The physiological ciliary structure consists of two components: a hairy structure that is capable of being distorted when exposed to force, and nerve cells (dendrite) that can detect this distortion.^[^ ^14^, ^15^ ^]^ This combination makes the ciliary structure exceptionally sensitive to even the smallest changes in its surrounding environment.^[^ ^9^, ^10^, ^11^, ^12^, ^13^ ^]^ Therefore, the flexibility of ciliary mechanosensors allows for a wide range of potential applications, from detecting airflow,^[^ ^16^, ^17^ ^]^ water flow,^[^ ^18^, ^19^ ^]^ and recognizing their environment^[^ ^20^ ^]^ for robots. Current artificial cilia‐based sensors rely on piezoresistivity, capacitive, and magnetic stray field sensing mechanisms. Piezoresistive sensing involves creating changes in the electrical conductance of a substrate upon compression or strain when a cilium is bent from physical perturbation such as under the influence of air or liquid flow.^[^ ^21^, ^22^ ^]^ Capacitive sensing relies on the disturbance of the cilia inducing physical changes on the underlying substrate, differentially increasing or decreasing the gap between capacitive plates and modifying capacitance.^[^ ^19^, ^23^ ^]^ Magnetic sensing utilizes a magnetically polarized cilium, which when bent induces a stray field that is detectable by magnetic sensing substrates.^[^ ^24^ ^]^ Fabricating these micro‐sized cilia is commonly done through templates,^[^ ^25^ ^]^ micro‐electromechanical systems,^[^ ^26^ ^]^ self‐assembly,^[^ ^27^ ^]^ cilia pulling,^[^ ^28^ ^]^ and laser sintering‐based printing.^[^ ^29^ ^]^

The inclusion of cilia‐like protrusions within the elastomer enables environmental responsiveness, allowing sensors to interact with and navigate their surroundings. To enhance the performance of e‐skin‐based mechanosensors, three key aspects need to be considered: i) a simple and multifunctional sensing mechanism, ii) scalability and customizability, and iii) sensing linearity. However, existing sensing mechanisms are often limited to a specific target application, such as tactile, airflow, or liquid flow sensing. The contact‐resistance‐based sensing method offers the advantages of multifunctional sensing, low operation voltages, and a simple device architecture.^[^ ^30^, ^31^ ^]^ In this method, when the cilia connected to the source electrode are bent by a stimulus and contact with the cilia connected to the drain electrode, a change occurs in the current flowing between the electrodes. This mechanism enables two modes of sensor operation: dynamic mode, where moving cilia interact with a stationary substrate, and static mode, where external agents deflect stationary cilia. Acting as an on‐off switch, this inter‐cilium contact method triggers a transition in current from an initial “off” state to an “on” state when the stimulus crosses a specific threshold. This capability is particularly effective in applications requiring precise detection of the moment when the stimulus exceeds the threshold. The simplicity of this on‐off sensing mechanism extends its applicability beyond single applications like airflow, liquid flow, and tactile sensing. It can be applied in bioelectronics, such as artificial eyelashes that respond to mechanical stimuli like dust and airflow simultaneously, as well as in tactile sensing in both liquid and dry environments or whiskers that require dynamic sensing to explore a space and static sensing to perceive nearby stimuli.^[^ ^20^ ^]^ For instance, it can detect dust landing on a set of eyelashes, monitor hazardous increases in wind speeds, or identify significant defects on apparently smooth surfaces when scanned by a brush.

The bulkiness and lower aspect ratio of current cilia sensing setups could lend themselves poorly to applications that require a tight grouping of tens of high aspect ratio sensing cilia. For instance, human eyelashes have an average diameter of ≈61–71 µm and an average length of ≈8–12 mm with an associated aspect ratio of ≈154.^[^ ^32^ ^]^ To replicate the functionality of human eyelashes, it is essential to develop cilia‐like structures with high aspect ratios capable of blocking ultraviolet light, redirecting airflow, and triggering an electrical response upon being bent by external stimuli like dust.^[^ ^32^, ^33^, ^34^ ^]^ Consequently, simplicity in fabrication is desired for producing cilia arrays with high aspect ratios, allowing for shorter production times, reduced environmental harshness, and lower chemical costs. However, previous cilia‐based sensors that employ contact‐based sensing typically possess diameters ranging from 120 µm to 1.5 mm and lengths ranging from 1 to 8 mm, resulting in lower aspect ratios.^[^ ^24^, ^35^, ^36^, ^37^, ^38^ ^]^ Since the sensitivity of cilia to bending under a uniform force or pressure depends on the aspect ratio, customization options during fabrication are crucial to achieve desired sensitivity ranges. Furthermore, cilia‐based sensors utilized in airflow, water flow, and tactile sensing often exhibit sigmoidal growth with a distinct plateau in sensor response within a narrow hyper‐sensitive range, a feature known as sensing non‐linearity.^[^ ^6^, ^24^, ^38^, ^39^ ^]^ This is mainly due to the isotropic nature of cilia structures or arrays. Therefore, devices that incorporate non‐uniform parameters such as variable inter‐cilia spacings, lengths, or diameters have the potential to offer high sensitivity and a wide linear response region, enabling the detection of a diverse range of stimuli.

Extrusion‐based 3D printing offers extensive flexibility and customization, allowing precise control over individual cilia and overall sensor design.^[^ ^40^, ^41^, ^42^ ^]^ Unlike traditional sensors with limited stimulus ranges, 3D‐printed cilia enable adjustment of their number, length, and diameter to modify sensitivity. This feature not only allows for the creation of a single linear sensor capable of detecting a wide range of stimuli but also facilitates the production of customizable artificial cilia sensors with adjustable sensitivity. Previous studies have explored 3D printing magnetic cilia that exhibit coordinated metachronal motions, as well as silicone elastomer‐based structures incorporating graphene resistive elements for tactile and water stimuli.^[^ ^38^, ^43^ ^]^ However, the concept of a fully conductive, 3D‐printable high‐aspect ratio cilia array sensor remains largely unexplored, as existing studies primarily focused on specific applications such as magnetic mixing and propulsion.^[^ ^44^ ^]^

Here, we present polycaprolactone (PCL)/graphene cilia sensing arrays that combine the control and ease of fabrication of 3D printing with contact‐resistance‐based sensing. These arrays enable the fabrication of highly customizable artificial cilia with a wide range of lengths (1–20 mm) and diameters (100 µm to 1 mm), providing fine control and tunable high aspect ratios (up to ≈200). By printing cilia connected to silver electrodes on a flexible adhesive substrate with a rubber layer, we achieve precise control over sensor geometry, accommodating various target sizes and contours. With this level of customizability, we present sensors which when taken together offer a wide linear response range which is impossible for smaller sensors with inflexible fabrication methods. Traditional cilia sensing archetypes are well explored in the cases of airflow sensing, water flow sensing, and tactile sensing, but remain unexplored for the cases of artificial sensing eyelashes and inter‐surficial motion sensing. The presented 3D‐printed artificial cilia arrays have a wide breadth of suitable applications owing to their ease of fabrication and simple sensing method.

## 2 Results and Discussion

### 2.1 3D Printing Strategy of Cilia Sensors

To print high‐resolution, free‐standing, and high aspect‐ratio cilia structures, a printable ink that is both conductive and rapidly cured is required. A PCL/graphene composite fills this niche well; graphene acts as a conductive nanofiller to form a well‐dispersed conductive network, while PCL is an easily accessible polymer with favorable mechanical properties and high impact resistance. As graphene is hard to disperse within polymer matrices, determining a co‐solvent for graphene and PCL is important. One framework to determine nanofiller‐polymer interactions is that of Hansen solubility parameters.^[^ ^45^ ^]^ The Hansen solubility parameters of PCL are [17.0 (dispersive), 4.8 (polar), 8.3 (hydrogen bonding)],^[^ ^46^ ^]^ while those of graphene are [18.0, 9.3, 7.7].^[^ ^47^ ^]^ Therefore, dichloromethane (DCM) was selected as an accessible solvent, with solubility parameters of [17.0, 7.3, 7.1] that are comparable to both graphene and PCL.^[^ ^48^ ^]^ This similarity in parameters may be explained by the large, polarizable chlorine atom in DCM, which provides the London dispersion forces capable of solubilizing graphene (2D hydrophobic carbon sheets) and PCL (long polymeric chains).^[^ ^48^ ^]^

**Scheme 1:**
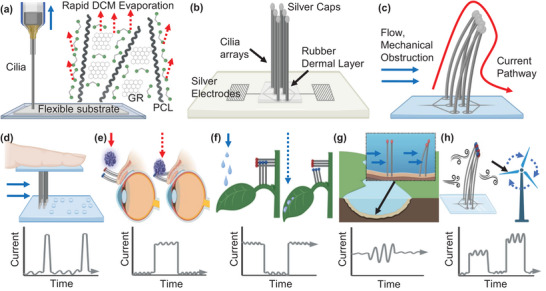
Schematic of printing scheme and sensing mechanism. a) Rapid DCM evaporation from a graphene/PCL nanocomposite ink enables the formation of high‐aspect‐ratio cilia. Printing is performed on a flexible substrate. b) An array of cilia is printed onto a silver conductive network. Silver caps are added to cilia tips to enhance sensitivity to stimuli. A rubber dermal layer improves the attachment and durability of cilia. c) Perturbances to the cilia cause the silver caps to come into contact, creating a pathway for current; this acts as the primary sensing mechanism. d) An active braille sensor detects small, raised features and may one day be attached to a human/robotic fingertip. The current remains effectively zero until a feature is scanned, resulting in a sharp peak. e) Artificial eyelashes to detect dust and debris may one day be a treatment for those with alopecia. The current remains effectively zero until debris deflects the eyelashes together, and it persists until the debris is removed. f) Inter‐surficial cilia may form a closed circuit at rest and separate upon disturbance, with possible applications in detecting hinge‐like motion or precise movement. The current initially has a non‐zero value until the cilia are bent apart, breaking the connection. g) Cilia sensors are waterproof and responsive to changes in water currents, offering the potential for environmental and industrial applications. The current fluctuates as the cilia deflect toward and away from each other during water flow. h) Airflow sensors may find use in climate measurements, automobiles, and home appliances. The current is zero until sufficient airflow bends one cilium into contact with the other. The magnitude of the current is proportional to the contact area and the time between cilia, which is related to the airspeed.

The PCL, DCM, and graphene are combined to form a conductive, homogenous paste, which is transferred into a pressurized syringe and extruded through a nozzle with inner diameters ranging from100 µm to 1 mm (**Scheme** **1**a). The step‐by‐step printing process is shown in Figure S1 and Movie S1, Supporting Information. The DCM solvent evaporates rapidly as the ink is extruded, and the ink hardens almost instantaneously, in a process known as solvent cast 3D printing. This rapid curing mechanism creates straight, high aspect‐ratio cilia if the nozzle is continuously moved upward while extruding.

The components of the sensor include electrodes, cups, cilia, dermal layer, and caps (Scheme 1b). Before printing an array of cilia, a conductive pathway of silver epoxy is first printed on the desired surface, forming two square electrodes connected to an array of circular “cups.” The cilia are printed so as to fill the cup, creating a current pathway between the cilia and the silver electrodes while still allowing the PCL/DCM composite to adhere strongly to the flexible substrate. Printing the cilia directly onto the silver epoxy without a cup or into a cup that is too small does not promote good adhesion and the cilium will not stay firmly rooted on the substrate (Movie S2, Supporting Information). To make the sensor more versatile, a thin layer of rubber is printed at the base of the cilia to form a rubber “dermal” layer to bind the cilia in place and diffuse mechanical stress from stimulus on the cilia. Small “silver caps” may also be added manually to the top of the cilia for a 2 × 3 (two rows of three cilia) or 1 × 2 array of thin cilia. This is necessary for the sensing mechanism in arrays with few total cilia as the cilia need to make pronounced, continuous contact with one another (Movie S3, Supporting Information). However, larger arrays of 10 × 10 cilia do not require the caps as sliding past the nearest adjacent cilium in sequence ensures contact with any of the other adjacent cilia (Figure S2, Supporting Information). While caps are beneficial for ensuring contact in smaller proof‐of‐concept devices, they are unnecessary for larger arrays comprising tens or hundreds of cilia. The cilia quickly become stiff within a few seconds of printing as the solvent evaporates, rendering the introduction of a silver cap irrelevant to their straightness. We have also experimented with printing these caps directly, but controlling their adhesion to the PCL graphene cilia is difficult.

### 2.2 Inter‐Cilium Contact Sensing

We introduce a novel sensing approach that utilizes a minimum of two conductive cilia that are initially separated. When the cilia sensor is subjected to air, water flow, vibration, or any other type of mechanical disturbance, the ends of the two cilia contact, forming a closed circuit and resulting in a change in the current flow (Scheme 1c). This detection method provides both dynamic and static capabilities, and its scalability and simplicity make it suitable for a variety of industrial, biosensing, and environmental applications.

Scheme 1d–h presents five examples of applications of cilia sensors and their pseudo‐graphs demonstrating a generalized version of current output for each sensing mode. In each mode, there is some initial current value, which is altered by stimulus bending the cilia with respect to one another. This change in current under a constant voltage application forms the foundation of each sensing method. 1) We call the framework of manipulating an array of cilia across a stationary surface “dynamic sensing” and demonstrate its proof of concept in a tactile braille sensor (Scheme 1d). This sensor has the ability to detect small features and act as a brush that can drag along a raised surface. The remainder of the applications are in “static sensing,” where a stimulus is introduced externally to stationary sensing cilia. 2) One of these applications is a cilia sensor that models human eyelashes (Scheme 1e). This sensor is able to detect if a force, such as that of falling debris, is placed onto the cilia/eyelashes. Such sensors may be useful in robotics, and even pursued as a treatment for those with alopecia. 3) The third configuration (Scheme 1f) consists of two arrays of cilia on perpendicular substrates embedded within one another, forming a closed circuit. When the two substrates move apart from one another, the conductive pathway is broken (open circuit). This type of sensing is referred to as “inter‐surficial” motion and may be modeled after how a leaf bends from rainfall or from the landing of an insect. 4) The fourth configuration (Scheme 1g) is another static sensor type to measure water flow. Unlike sensors exposed to the air, the innate electrolytes in water create a closed circuit without the cilia touching. The flow of water brings the cilia closer together, reducing the length of the pathway between cilia and increasing current through the system. 5) Last, a cilia array is designed to detect changes in airflow (Scheme 1h). This design features cilia of different thicknesses, with the thinner cilium displaying higher sensitivity, and bending further than the thicker cilium in response to airflow perturbations. This asymmetry in bending sensitivity prevents the cilia from bending at similar distances and not coming into electrical contact. Overall, these sensors are highly versatile in their potential by incorporating a mechanism reliant only on inducing electrical contact between conductive cilia. The ink composition, and the spacing, size, and conductivity of each cilium can be easily customized through 3D printing and a broadly accessible ink fabrication process.

### 2.3 Characterizations of 3D Printable Inks

**Figure 1:**
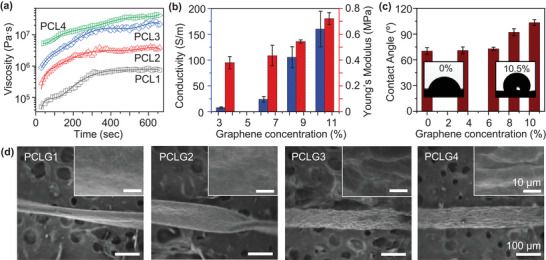
a) Using rheology, the viscosity of four PCL composites was measured during the curing process. These composites exhibit a nearly linear increase of viscosity followed by an asymptotic plateau when the curing process is complete. The curing time and the corresponding viscosity increase with PCL concentration. b) Conductivity and Young's modulus are seen to increase with increasing graphene concentration, showing the composites become more rigid and more conductive. c) Contact angle of PCL (0% graphene concentration), PCLG1 (3.5%), PCLG2 (6.5%), PCLG3 (8.5%), PCLG4 (10.5%). The wettability of the cilia in a sensor can be tuned with graphene concentration for applications in water or humid environments. d) SEM images of PCLG composites. The surface roughness increases markedly with increasing graphene concentration, and the general cylindrical shape is retained best in concentrations with higher graphene volume fractions.

The ratio of PCL/DCM was first iteratively studied in order to optimize the curing time, conductivity, and stiffness of the PCL/DCM/Graphene blend. Four PCL/DCM blends were created at PCL weight percentages of ≈10%, 20%, 30%, and 40%, labeled PCL1, PCL2, PCL3, and PCL4. Stress–strain experiments were conducted on cylindrical samples of the ink, which revealed that the Young's moduli increased with increasing PCL concentration to ≈3.88, 7.02, 8.73, and 15.95 MPa, respectively (Figure S3, Supporting Information). Rheological measurements were also performed to measure viscosity over a 10‐min period at a constant shear rate and demonstrated that the inks cured at slower rates and over longer time scales with increasing PCL concentration (**Figure** **1**a). The data revealed a nearly linear increase for over 300 s, followed by an asymptotic plateau that indicated curing. With increasing PCL weight percentage, the change in viscosity per unit time decreased, and the time at which full curing was observed increased. To achieve successful extrusion‐based direct ink writing of freestanding cilia, the prepared ink must be viscous enough to maintain its shape after printing. Additionally, the ink should cure or solidify at the same rate as it is being extruded during the printing process. This synchronization ensures proper formation and structural integrity of the printed cilia. For printing purposes, the PCL/DCM blend with the optimal uncured stiffness to hold shape during printing and a curing time similar to the rate of extrusion was selected to be PCL3.

Graphene was then iteratively introduced into the ink at weight percentages of ≈3.5%, 6.5%, 8.5%, and 10.5%, and labeled PCLG1, PCLG2, PCLG3, and PCLG4 at the fixed PCL/DCM ratio of PCL3. As the graphene concentration in the blends increased, the conductivities and Young's moduli of the composites also rose, reaching ≈8, 24, 105, and 160 S m^−1^, respectively, and ≈0.38, 0.43, 0.54, and 0.72 MPa, respectively (Figure 1b) (Raw data are shown in Figure S4, Supporting Information). These results were further confirmed through Raman spectra and X‐ray photospectroscopy (XPS), which showed that the composites were both stiffer and more conductive (Figure S5, Supporting Information). The hydrophobicity of the PCL/DCM/Graphene composites was also tested via contact angle measurements in order to determine their suitability for sensing applications in water and in humid environments (Figure 1c). The contact angles of PCL without graphene, PCLG1, and PCLG2 are similar at roughly ≈70–72°, while those of PCLG3 and PCLG4 increase to ≈92–100°. The increased hydrophobicity at increased graphene concentrations reflects the adequate dispersion of the hydrophobic graphene nanosheets at higher concentrations.

Scanning electron microscopy (SEM) was used to examine the 3D‐microstructure of cilia printed at different graphene concentrations (Figure 1d). At lower concentrations, the higher weight fraction of DCM evaporating caused the cilium to twist and wrap around itself as it cured. Higher concentrations of PCL and Graphene resulted in a rougher, more cylindrical surface. It was found that an intermediate concentration of PCL/DCM/graphene (PCLG3) displayed good mechanical properties, high conductivity, and cured at the desired time frame without twisting. The cilia were printed on a one‐sided adhesive elastomer substrate, with the adhesive end allowing attachment to various surfaces. Moreover, the rubberized backing on which the cilia were printed favorably interacts with DCM to improve adhesion (Figure S6, Supporting Information). At the onset of printing, the DCM within the ink dissolves the nonpolar rubber substrate, allowing the ink to diffuse slightly and bind to the substrate well upon DCM evaporation. To ensure the cilia were straight, the rate of evaporation, rate of extrusion, and rate that the nozzle tip was raised were balanced such that the material was extruded and hardened at the same rate the nozzle tip moved up from the substrate.

### 2.4 Characterizations of 3D‐Printed Cilia Arrays and Sensors

**Figure 2:**
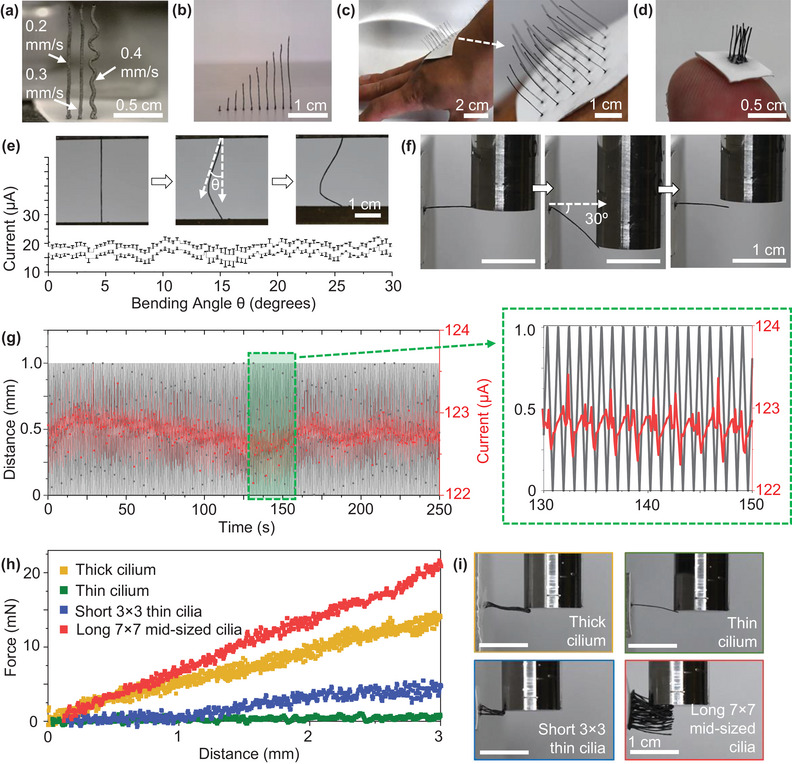
Cilia and sensor characterization. a) Cilia straightness as a function of printer speed. b) Increasing cilia heights from 1 to 20 mm at intervals of 2 mm. c) 5 × 5 cilia array [diameter (*d*) = 100 µm, length (*l*) = 2 cm] as a demonstration for a larger scale device wearable or implantable on a surface the size of a hand. d) 3 × 3 cilia array (*d* = 100 µm, *l* = 0.5 cm) as a demonstration for a smaller scale device wearable or implantable on a surface the size of a fingertip. e) Piezoresitivity test. As a cilium is bent to 30 degrees of its original shape and current is passed from one of its ends to the other. The current through the cilium is unchanged, suggesting no piezoresistive behavior and a good candidate for an inter‐cilium contact‐based sensor. f) Bending restoration test. After being bent 30°, a cilium (*d* = 100 µm, *l* = 1 cm) restores to a few degrees from its original shape under the prolonged torque from gravity. g) Cyclability test of a cilium bent toward another (*d* = 100 µm, *l* = 1 cm for both) in DI water for 250 cycles (1 cycle takes 1 s) as the current between the cilia is measured with a constant 2 V bias. h) Force sensitivity comparison. Four types of cilia are bent 3 mm by a texture analyzer: single thin cilium (*d* = 100 µm, *l* = 1 cm), a 3 × 3 array of thin cilia (*d* = 100 µm, *l* = 0.5 cm), a single thick cilium (*d* = 1 mm, *l* = 1 cm), and 7 × 7 array of mid‐sized cilia (*d* = 500 µm, *l* = 1 cm) in order of increasing stiffness. i) Photo images from the four types of cilia bending measurements.

Adjusting the dispensing pressure or nozzle tip moving speed during extrusion controls the straightness of the cilia (**Figure** **2**a). Incorrect synchronization of ink extrusion and nozzle movement results in over‐extrusion, leading to longer path lengths and the formation of curly cilia (0.4 mm s^−1^), or rapid collapse of the ink due to gravity before curing (0.2 mm s^−1^). These parameters are influenced by ink viscosity, printer speed, and nozzle diameter. When proper speed control is achieved, cilia lengths ranging from 1–20 mm can be printed by changing pathways dictated by a simple G‐code command (Figure 2b).

To demonstrate the versatility of the fabrication process, we printed two cilia (100 µm diameter) sensors without the silver electrodes. The first array was a 5 × 5 arrangement with a length of 20 mm with 7 mm inter‐cilia spacing (Figure 2c), while the second array was a 3 × 3 arrangement with a length of 5 mm with 1 mm inter‐cilia spacing (Figure 2d). Remarkably, both arrays were printed using the same printing parameters, with the only difference being the addition of two extra cilia per row and per column in the larger array. It is important to note that the printing parameters significantly affect the response and target of the cilia. For instance, a sensor designed to be mounted on a drone may require different considerations compared to one intended for a wind turbine or a river instead of a small water pipe. Hence, our 3D printing offers a flexible fabrication method for creating sensors tailored to various scales, providing incredible customizability and avoiding a one‐size‐fits‐all approach.

To assess the impact of direct bending on the conductivity of the cilia, we conducted tests to measure their current as a function of bending angles under constant 2 V bias (Figure 2e). Measured current values were not significantly affected by piezoresistive effects typically observed in strain sensors, indicating that contact‐resistance sensing is the key mechanism at play, rather than piezoresistive sensing. This finding affirms the suitability of the cilia in harsher conditions where piezoresistive effects from the environment are not desired.

To further evaluate the durability of these sensors in harsh environments, a series of tests were conducted. First, a 1 cm long and 100 µm wide cilium was bent 5 mm (30°) beyond its neutral position. The cilium quickly restored to within 500 µm (2.5°) of its original state within a few seconds. This restoration process was observed even when subjected to prolonged torque from gravity (Figure 2f and Movie S4, Supporting Information). Next, to ensure the reliability of the fully fabricated sensor, a cyclability test was performed for water flow sensing. The test involved 250 cycles where the electrical current between a 2 × 1 array of cilia measured as one cilium was deflected toward the other using a programmed, measured displacement through a programmable texture analyzer (Figure 2g). The results showed stable and cyclical current readings, demonstrating consistent device performance even after numerous cycles and when fully submerged in water.

Furthermore, to evaluate the sensor's resistance to corrosion when exposed to water for an extended period, two sets of silver electrodes were subjected to a voltage sweep ranging from −2 to 2 V. One set of electrodes (control) was kept in the air, while the other (experimental) was submerged in water for a week (Figure S7, Supporting Information). Remarkably, the resulting current–voltage (*I*–*V*) curves were nearly identical for both sets of electrodes. This observation suggests that the silver epoxy electrodes effectively resist corrosion even after prolonged water exposure.

To investigate the sensitivity of cilia to bending, the thickness (diameter) of a cilium and the number of cilia in an array play a crucial role and determine their suitability for different applications. To understand the impact of these variations, we conducted experiments with four different types of 3D‐printed cilia and measured the bending force as a function of the bending distance (Figure 2h,i). Four types of cilia were tested: a single thin cilium (100 µm diameter, 1 cm length), a 3 × 3 array of thin cilia (100 µm diameter, 0.5 cm length), a single thick cilium (1 mm diameter, 1 cm length), and a 7 × 7 array of mid‐sized cilia (500 µm diameter, 1 cm length). For our analysis, we considered an inter‐cilia spacing of 1 mm. When a cilium bends 1 mm toward its nearest neighbor, it makes contact, allowing current flow. Through comprehending the precise amount of force necessary to bend various types of arrays at a specific distance, we acquire the ability to regulate the moment at which the cilia make contact with one another. In most of the devices we printed, such as the braille scanning, eyelash simulation, and airflow sensing applications, the cilia were designed with a 1 mm inter‐cilia spacing. Therefore, the specific force needed to induce electrical contact is determined by the force required to bend the various cilia to 1 mm. Our findings showed that for the presented orientations with a 1 mm inter‐cilia spacing, the force required to induce electrical contact was less than ≈7.5 mN in each case. Notably, a single 100 µm diameter cilium exhibited the lowest force requirement of ≈0.35 mN. This indicates that by adjusting the spacing, array size, and cilium thickness, we can precisely control the necessary force to achieve electrical contact. This flexibility allows us to tailor the sensor array to specific stimulus parameters. Applications requiring high force sensitivity can utilize a few 100 µm cilia, while those involving larger forces can incorporate more cilia with increased thicknesses (diameters). To better understand the bending behavior of cilia and enable precise design of arrays with specific stimulus parameterization, we can establish a theoretical framework that comprehends the bending shape exhibited by the cilia.

### 2.5 Design of Dynamic Sensors by Euler–Bernoulli Beam Theorem

The advantages of a 3D printing process permit us to manufacture cilia sensors with predetermined physical dimensions that are optimized for specific applications. By using Euler–Bernoulli beam theory to model the response of cilia subject to point forces and linear pressures along the length of the cilium, we can customize the design of the cilia to accurately detect stimuli of a predetermined size or strength. To validate the bending behavior of the fabricated cilia under different parameters, we utilize an analytical treatment and employ finite element analysis (FEA) to simulate the resulting beam shapes. Once the bending behavior is validated, particularly in scenarios involving obstruction from raised features such as dynamic sensing, we establish a model that parameterizes the cilia's length, spacing, and dimensions of the target objective. With this model, we can design cilia‐sensing devices tailored to the specific predicted stimulus when scanning a system with known width and height features. Additionally, the ciliary structure can be adjusted in terms of diameter, length, and inter‐cilia separation to optimize its response to a raised dot while disregarding features below a quantifiable threshold.

We first examine a cantilever's response to bending under a uniform pressure, as would be observed in air or water flow. The general expression for a cantilever beam of elastic modulus *E*, moment of inertia *I*, and length *L*, bending in response to a linear force density *ω* deflects an amount *y* at a point *x* along the beam:

(1) $$y=\frac{\omega x^{2}}{24EI}\;\left(x^{2}+6L^{2}-4Lx\right)$$

The second category of bending type is in response to a point force *F* at a position *a* along the cantilever, as would be observed in the tactile sensing application. For this point force, the bending of the cantilever is broken into two distinct regions:

(2) $$y\;=\frac{Fx^{2}}{6EI}\;\left(3a-x\right),\;\mathrm{for}\;0<x<a$$

(3) $$y\;=\frac{Fa^{2}}{6EI}\;\left(3x-a\right),\;\mathrm{for}\;a<x<L$$

**Figure 3:**
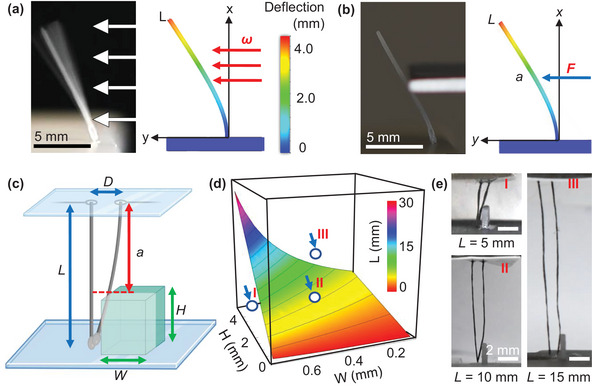
Euler–Bernoulli beam theory analysis for cilia bending. a) Experimental and simulated analysis of a 100 µm‐thick, 10 mm‐long cilium bending under 1150 Pa pressure from airflow. [The cilium undergoes complete oscillations as the air bends it beyond its equilibrium position, and the material's restoring force then bends it back through equilibrium within the brief duration of the camera shutter being open.]. b) Experimental and simulated analysis of the same dimension cilium bending under 1.9 mN of force at 5 mm along its length. c) Schematic cilium of length *L* and separation *D* undergoing scanning at point a by a feature of width *W* and height *H*. d) A surface map of the cilia length necessary to induce contact while scanning a surface feature with variable width and height. I) First test case in the 3D space with variables *L* = 5 mm, *W* = 0.45 mm, *H* = 2.5 mm, which rests below the surface and induces bending, which is overly sufficient for contact. II) Second test case of variables *H* = 15 mm, *W* = 0.45 mm, *H* = 2.25 mm, which rests directly on the threshold surface and only induces contact between cilia. III) Third test case of variables *L* = 10 mm, *W* = 0.75 mm, *H* = 3.5 mm, which rests well above the surface and creates insufficient bending for contact.

To verify the models experimentally, a cilium made of PCL dissolved in DCM, without any nanofiller, is printed at 10 mm tall through a 100 µm diameter nozzle. The 3D‐printed cilium is then subjected to a constant linear force of 115 mN m^−1^ from a controlled pressure dispenser set to 1150 Pa (**Figure** **3**a). This results in a maximum deflection at the equilibrium position of 4.30 mm at point *L* at the tip of the cilium. To further corroborate this model, FEA of a cylindrical beam of identical dimensions with an identical linear force along its length is performed, yielding a maximum displacement of ≈4.23 mm, which closely matched the deflection predicted by our model.

The sensitivity of the cilium to a point force is measured experimentally by applying a force at point *a* = 5 mm along its length, where the responsive force *F* is measured by a texture analyzer as it deflects (Figure 3b). The maximum deflection of the end of the cilium is *y* = 4.46 mm, while the midpoint of the cilium is *y* = 3.00 mm with a measured responsive force of 7.5 mN (Figure S8, Supporting Information). FEA of an identical cylindrical beam bound at one end with a point force at *a* = 5 mm returned a maximum displacement of ≈4.93 mm, in good agreement with the experimental results and with an identical bending shape. The bending profile from the experiment and simulation also matches the theory, which predicts the bending is distinctly non‐linear and cubic for *x* < *a* and linear for the region *a* < *x* < *L*.

Further analysis is directed to the case of a dynamic tactile sensor. Our proof of concept dynamic sensor is comprised of two adjacent cilia which contact one another when one of the cilia is bent by a feature, such as a braille dot (Figure 3c). Let the length of the two cilia be given as *L*, their unbent separation as *D*, and the surface feature's width and height as *W* and *H*, respectively. When the cilia contact the surface, a feature bends one toward the other at a point *a* (or *L − H)*. The point force the cilium is subjected to then is located at point *a* along the cilium. The tip of the disturbed cilium may or may not contact the other cilium depending on the extent of bending and the width of the feature. The following two conditions use the point‐bending equation presented above for a cilium deflected by a raised feature:

(4) $$y_{\max}=\;D\;=\frac{F{\left(L-H\right)}^{2}}{6EI}\;\left[3L-\left(L-H\right)\right]$$

(5) $$y_{L-H}=\;W\;=\frac{F{\left(L-H\right)}^{2}}{6EI}\;\left[3\left(L-H\right)-\left(L-H\right)\right]=\frac{2F{\left(L-H\right)}^{3}}{6EI}$$

In Equation (4), for the two cilia to touch one another, the tip at *x* = *L* must have deflected the full *D* separation. In Equation (5), at point *x* = *a*, or *x* = (*L* − *H*), the cilium has deflected the full‐width *W*. And so, we rewrite *D* in terms of *W* and rearrange to obtain four equations for each parameter of the surface features and cilium properties:

(6) $$2\left(L-H\right)\times D=W\left[3L-\left(L-H\right)\right]$$

(7) $$L\;=\frac{H\left(2D\;+\;W\right)}{2\left(D\;-\;W\right)}\;$$

The full system of equations (*H*, *D*, and *W*) is given in Equation S1, Supporting Information.

To test our system of equations, at a fixed inter‐cilia spacing (*D*), we plot three of the variables to form a surface plot that represents the minimum threshold for the contact between cilia given various cilia lengths (*L*), feature heights (*H*), and widths (*W*) (Figure 3d). These parameters are interrelated and can be used to calculate, for example, the length of cilium needed to detect a feature of known width and height, or the minimum height detectable of a feature with a known width by cilia of known length. We then choose three sample points in the 3D space to represent three distinct cases to demonstrate this principle (Figure 3e). The first sample point (Figure 3e‐I) is below the surface, which indicates overly sufficient bending, and the two cilia will touch beyond initial contact. The second sample point (Figure 3e‐II) is on the surface, indicating that the two cilia should just touch. Finally, the third sample point (Figure 3e‐III) is above the surface, which suggests insufficient bending, where the cilia do not touch. To verify the results, we print cilia of specified lengths and small rigid features with a stereolithographic printer to match the sample points. The cilia are made to contact the printed features at point *a* along their length. Each of the three cases behaves as the model predicts, demonstrating that designing a tactile sensor with theoretical models to guide parameterization is feasible.

Finally, we conducted FEA to investigate the impact of silver caps on the bending behavior of the cilia (Figure S9, Supporting Information). When subjected to a force of 10 µN, cilia with and without silver caps exhibited bending distances of ≈4.0 and ≈2.5 mm, respectively, indicating a significant difference in their response. For applications involving scanning mode (dynamic sensing), the addition of caps is expected to have minimal effect on the bending shape of the cilia. In scanning mode, the cilium is bent to a specific distance determined by the width of the feature, and the point of bending is determined by the height of the feature. In other words, the cilium will bend a set width determined by the surface feature regardless of its own stiffness. However, when considering theoretical models that predict the bending behavior of these cilia under known applied forces (static sensing, like from airflow, water flow, bending from dust, etc.), the presence of caps alters the moment of inertia and becomes significant in accurately characterizing their shape. Taking this framework into account, we can design sensors tailored specifically for applications such as surface mapping and braille reading, where precise bending characteristics are essential.

### 2.6 Dynamic Sensing: Surface Mapping for Braille Reading

**Figure 4:**
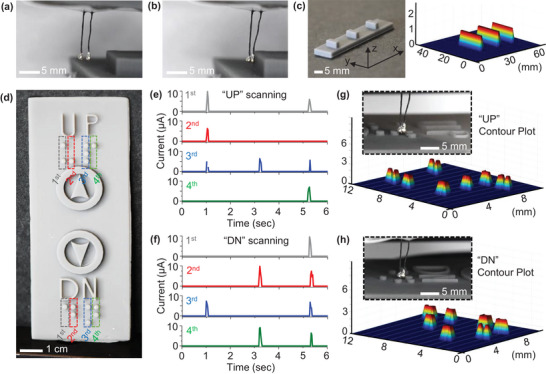
Dynamic sensing applications. a) When not in contact with a feature, 2 × 1 array of cilia does not bend, creating an open circuit. b) 2 × 1 array contacting a surface feature close the circuit and create a current pathway between cilia. c) 3D surface map created from compositing multiple scans in the *x*–direction at different positions in *y* and constant position in *z*. d) Elevator button was modeled with CAD and printed with braille lettering for UP and DN. e,f) Scan for the third column of the UP and DN Braille with distinct current peaks for each Braille dot at consistent points in time. g,h) Contour plot of the composited UP and DN scans produces an accurate map of the braille cells.

A proof of concept was printed with a simple structure containing three prismatic surface features. To test the device, a 2 × 1 configuration was used (**Figure** **4**a). If the adjacent cilia touched, the circuit would close, allowing electrical current to flow (Figure 4b). Silver “caps” were added to the tops of the cilia to ensure good contact interface. Again, with a large sensor containing many adjacent cilia (e.g., ≈10 × 10 array), caps may not be necessary due to induced contact between non‐adjacent cilia (Figure S2, Supporting Information). The cilia sensor was attached to a gantry‐controlled motion stage, and made to scan the 2 × 1 sensor five times at a constant speed in the *x*–direction. Each trial was evenly spaced on the *y*‐axis, with the *z* position of the cilia held constant. Using a simple coding process (Figure S10, Supporting Information), current data under a constant 2 V bias as a function of time was translated into a 3D plot of features at specific *x* and *y* coordinates (Figure 4c). The program recognizes a data point corresponding to a raised feature if it meets either of the following criteria: the data point is in the range of >≈1 µA or has a first‐time derivative of >≈1000 A s^−1^. Any significant spike or sustained current in the µA range or larger is considered to be due to contact between the cilia. It is important to note that different types of stimuli, varying numbers and orientations of cilia, and different time scales can result in different operating current ranges for the cilia. In this particular case, we consistently observed that a minimum operating current of ≈1 µA or a first‐time derivative greater than ≈1000 A s^−1^ reliably indicated a signal from the cilia touching, distinguishing it from noise. The 3D plot produced from the scan of these three features was accurate and represented the physical geometry of the printed structure accurately.

An e‐skin‐inspired device equipped with a dynamic sensor constructed from dozens of adjacent cilia, attached to a fingertip, may prove highly useful in braille reading. The size of the Braille characters is standardized at a height of ≈0.9 mm, a base diameter of ≈1.5 mm, and a dot spacing of ≈2.5 mm. As a proof of concept, an elevator button with braille lettering for the letters UP and DN was modeled (Figure 4d). The inter‐cilia spacing was set to 1 mm, and using the aforementioned equations, the expected stimulus from the braille dots was calculated to produce a cilia length of ≈10.0 mm. To test the sensor, two sets of scans were conducted for the UP and DN braille characters (Figure 4e,f). The scans had consistent current readings and even spacing between current peaks, given the constant scanning speed. The scans were then compiled and modeled in 3D space to produce two high‐resolution 3D plots of the braille patterns (Figure 4g,h). The contour plot suggests that the device is suitable for modeling more complex surfaces with non‐binary *z* heights in a future study. The overall maximum forces of ≈27.5 mN and the sensitivities of ≈57 µA mN^−1^ were observed for 1 mm diameter cilia, which is compatible with previously reported cilia‐based tactile sensors of similar hair‐like dimensions (Table S1, Supporting Information).^[^ ^24^, ^36^, ^37^ ^]^

### 2.7 Static Sensing: Bioinspired Eyelashes, Flow Rate, and Inter‐Surficial Sensor

**Figure 5:**
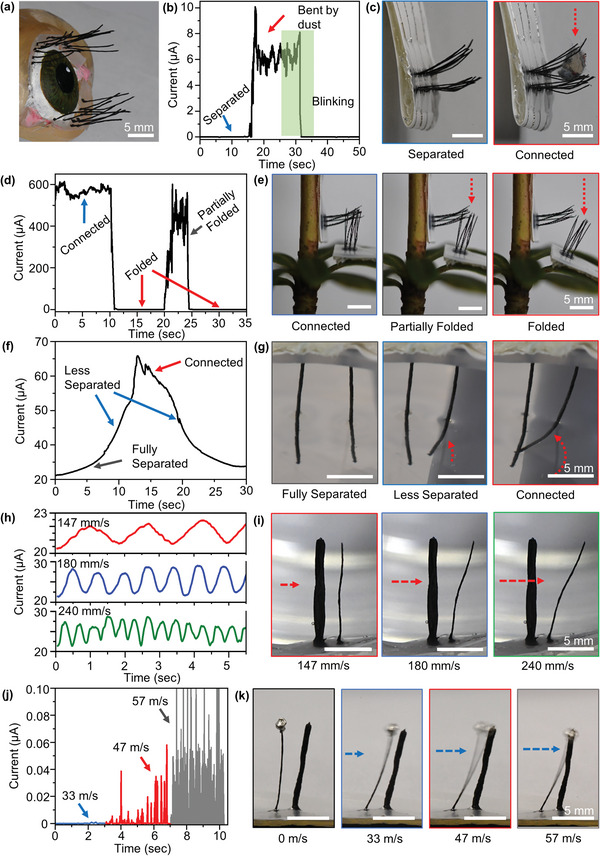
Static sensing applications. a) Simulacrum of human eye with cilia directly printed atop the eyelid as proof of concept. b,c) Two rows of printed eyelashes on a single eyelid connected to electrodes and unbent. Current–time graph of the eyelashes as they undergo bending from a piece of dust, forming an electrical connection. This creates an electrical signal which induces blinking to remove the dust. d,e) Inter‐surficial motion between two cilia arrays held 90° from one another on a plant stem and leaf. As the leaf, and its attached array, bends away from the stem, the electrical connection is broken, and the cilia become un‐embedded. A current–time graph is given for a leaf connected, fully bent, and partially bent. f,g) Cilium bending at constant speed toward an unbent cilium in DI water creates a steadily increasing peak because the autoionization of water lends it a small amount of inherent conductivity. h,i) Modified water flow sensor. Stiff and thin cilia were placed in DI water and stirred perpendicular to their orientation. Variable stirring rate induces variation in amplitudes and frequencies of current oscillation. j,k) The aforementioned orientation of a stiff cilium and thin cilium were subjected to variable airflow.

The alternative to the dynamic sensor modality is the static sensor. Static sensors do not actively stimulate a surface, but passively respond to stimuli, ranging from dust and debris to inter‐surficial motion to water flow and airflow.^[^ ^49^, ^50^ ^]^ An example of a static sensing application is the use of artificial eyelashes (**Figure** **5**a) (Movie S5, Supporting Information). Artificial eyelashes, consisting of an array of 9 × 2 cilia, are printed onto a flexible substrate to mimic a single eyelid with two rows of eyelashes (Figure 5b). During the sensing measurement, a steady 2 V bias was consistently applied across the electrode. When a piece of dust is placed onto the top row of artificial eyelashes, the cilia will bend downward and contact the bottom row of cilia, forming a current pathway (Figure 5c). When the debris is removed (blinking), contact between the rows of cilia is lost, and the cilia return to their default orientation.

Extensive research has highlighted the beneficial aerodynamic interactions of eyelashes with airflow, blocking ultraviolet (UV) light from the eye, and preventing particle deposition on the delicate eye surface below.^[^ ^32^, ^33^, ^34^ ^]^ The next generation of wearable biosensors could significantly benefit from artificial eyelashes that not only divert airflow and block sunlight, but also possess the ability to detect subtle deflections. Previous studies have explored the use of triboelectric nanogenerator‐based motion sensors to detect eyelash motion and transmit the information to a computer interface, enabling users to write simple computer programs solely through voluntary control over blinking.^[^ ^51^ ^]^ Additionally, Nishihashi et al. have demonstrated a wireless power transmitter for ultra‐small wearable devices, with a proof of concept involving attaching the receiving antenna module to an eyelash.^[^ ^52^ ^]^ These advancements highlight the integration of physiological cues with sensor‐based responses, opening up new possibilities for artificial eyelashes. Furthermore, these static sensors hold potential in various applications, including the development of sensitive airborne camera lenses within drone‐assisted camera networks.^[^ ^53^ ^]^ By leveraging the capabilities of static sensors, advancements in imaging technology and aerial surveillance can be achieved.

An alternate configuration of cilia is inter‐surficial sensing, which involves the sensing of the motion between two surfaces. This is demonstrated in Figure 5d, which shows the movement of a plant leaf with respect to its stem. In comparison to dynamic sensing and the eyelash applications mentioned previously, this type of cilia employs a closed circuit as its default state. The two arrays of 3 × 3 cilia are oriented 90 degrees from each other and embedded within one another, allowing for the current to flow (Figure 5e) (Movie S6, Supporting Information). When the leaf is bent away from the stem, either due to accumulated rainfall, an insect landing, or leaf growth, the cilia detach from one another and the current pathway is broken. Although flexible strain sensors are well‐suited for physiological sensing of bending, it is important to highlight the distinct advantages offered by cilia arrays.^[^ ^42^, ^54^, ^55^ ^]^ The proposed cilia sensors can be effortlessly integrated between two surfaces in different orientations, accommodating a wide range of inter‐surficial motions such as vibration, sliding, twisting, bending, and more, without any interference. In contrast, strain sensors often rigidly connect surfaces, impeding their ability to bend freely. Furthermore, unlike strain sensors that introduce rigidity and hinder bending, our cilia sensors establish non‐rigid physical contact. This non‐rigid contact allows the surfaces to maintain their inherent flexibility and responsiveness to stimuli. It preserves their ability to bend naturally, ensuring that the sensing mechanism does not compromise the overall flexibility of the system. Hence, this type of cilia sensing can be used to detect leaf wetness,^[^ ^56^ ^]^ plant growth,^[^ ^57^ ^]^ and plant health,^[^ ^58^ ^]^ and its versatility allows for multiple stimuli to be monitored simultaneously at tunable scales in environmental applications.

Static sensing can also be used to detect flow in both air and liquid. In this case, pressure is applied to the entire ciliary surface. As a result, both cilia are affected, causing them to oscillate. Due to the contrasting conductivity properties of water and air, the cilia exhibit no change in current when in an unbent state in air. However, when submerged in water, they exhibit a detectable latent flow of current. As a result, the sensor generates a measurable signal whenever the cilia bend relative to each other, not solely upon contact.

To understand the current flow between two cilia in deionized (DI) water, an experiment was conducted in which one cilium was manually deflected toward the other via a gantry unit. The results of this test showed that the scanning mechanism in water is functional, with a distinct peak present when the cilium was bent (Figure 5f). Additionally, this data revealed the operating current range as the distance between the cilia decreases. At 1 mm apart, ≈20 µA of current under a constant 2 V bias was observed to pass through the DI water (Figure 5g). As the distance between the nozzle tip of the bent cilium to the unbent cilium decreased, the current increased to over 50 µA, before dropping back to 20 µA when they were separated again. This data provides insight into the response of cilia to water flow and can be used to accurately determine the separation between the cilia, resulting in high‐resolution information about water flow strengths and rates.

When designing a functional water flow sensor, one challenge to consider is that two identical cilia are likely to displace equivalent distances when subject to the same fluidic pressure. 3D printing technology can be used to create two non‐identical cilia for an asymmetric design which increases sensitivity by only measuring the bending of a single sensitive cilium. One cilium is printed with a thicker, more stationary design, while the other is thinner and more deformable. This allows for bidirectional sensing, with the thin cilium bending toward the thicker in one flow direction and away from it in the other, with an associated shorter and longer current pathway, respectively. To test this modified flow sensor, it is placed into a beaker of water. A spherical probe is then attached to the Gantry unit and used to stir the water in circular motions at varying stirring speeds (Figure 5h,i). In order to determine the actual speed of water flow, stirring experiments were conducted at three different rates. To calculate the flow speed, a single drop of dye was introduced, and its path length was tracked over time by recording a movie. The measured water flow speeds were found to be ≈147, ≈180, and ≈240 mm s^−1^ for three distinct stirring rates, respectively. A detailed demonstration of this method can be observed in Movie S7, Supporting Information. The amplitude of the current oscillations varies with the frequency of stirring, with a higher stirring rate producing larger flow speeds and displacement of the cilia further past their unbent positions, thus increasing the current amplitude. This liquid flow sensing is well suited to various applications, such as lab‐on‐a‐chip^[^ ^59^, ^60^ ^]^ and traditional macro flow sensors,^[^ ^11^, ^18^, ^19^, ^38^ ^]^ due to its ability to detect micron scale deflections of the cilia from small flow rates, without requiring contact between cilium and the liquid.

Last, a two‐cilia system is used to test the sensitivity of airflow (Figure 5j,k). An asymmetrical system is 3D printed, with one cilium thinner than the other. Since air has a high electrical resistivity, the cilia must be close enough touch to induce current flow. To facilitate this, the thinner cilium is fitted with a “cap” to ensure contact if the cilia become misaligned. Air is expelled in a dispenser ≈5 cm away from the 2 × 1 array at pressures of ≈5, ≈10, and ≈15 psi, and the circuit current is monitored. The air speed was calculated from the outbound pressure assuming a conical spread of air resulting in a drag force on the cilium. The cilium was modeled as an upright beam that could bend at its base. Based on this model, the calculated air speeds correspond to the three pressures provided ≈33, ≈47, and ≈57 m s^−1^, respectively. At ≈33 m s^−1^, the thin cilium was deflected but did not bend sufficiently to contact the thicker cilium. At ≈47 m s^−1^, the cap on the thinner cilium began to collide with the thicker cilium, producing an irregular but clear current signal near ≈0.2 µA. At ≈57 m s^−1^, the capped cilia consistently collided with the thicker cilia, increasing the current output near ≈0.06 µA. The irregularity in the current output is due to small mechanical vibrations of the cilium, which arise from the interaction of the two cantilever beams in their own independent harmonic systems at high frequencies. The sensor's sensitivity to wind above a certain threshold is noteworthy. Air speeds lower than ≈33 m s^−1^ did not generate enough mechanical force for the cilia to contact. This property can be tailored for applications in which it would not be efficient to detect background airflow. In fact, this property allows for reverse engineering of cilia separation, radius, and length to target a certain wind speed threshold. Additionally, the asymmetric sensor displays anisotropy and enables directional sensing of airflow. Airflow that deflects the thinner cilium into the thicker cilium can be detected, while airflow in the opposite direction cannot. Multiple copies of these arrays, all of which are oriented at different angles with respect to one another, can be used to get spatially sensitive information about the precise direction of airflow. This opens up opportunities for completely customizable airflow measurements.^[^ ^6^, ^16^, ^24^, ^38^ ^]^

To demonstrate the range and sensitivity of our devices, we compared our results to previous ciliary‐based water and airflow sensors of similar hair‐like dimensions (Tables S2 and S3, Supporting Information). As a result of contact‐based sensing mechanisms, the airflow sensor's sensitivity (*∝* Δ*I*/*I*_0_) is ≈10^3^. This is several orders of magnitude greater than traditional sensors in which resistance changes incrementally with airflow. In addition, the cilia responded to maximum air speeds of ≈57 m s^−1^ compared to other works, which report between 7 and 30 m s^−1^, suggesting it can resist high wind speeds.^[^ ^6^, ^24^, ^38^ ^]^ Furthermore, the water flow sensors displayed a maximum water velocity of ≈240 mm s^−1^ and a sensitivity of ≈29 µA (m·s^−1^)^−1^, on par with previous ciliary sensors [max speeds from 66–100 mm s^−1^, sensitivities from 26–45.2 mV (m·s^−1^)^−1^].^[^ ^35^, ^38^, ^61^ ^]^ The strong mechanical properties of the 3D‐printed cilia impart a high airflow and water detection limit, allowing for sensor applications in harsh areas. Therefore, the robust mechanical properties of our 3D‐printed cilia enable high detection limits, enabling their usage in demanding environments. Moreover, our sensor design exhibits exceptional sensitivity, with distinct changes in electrical current that surpass sensitivities of ≈10^−4^ observed in other flexible cilia array sensors, where current shifts are small due to slight mechanical deformations.^[^ ^6^ ^]^ Overall, our sensors demonstrate superior performance, combining robust mechanical properties, high detection limits, and exceptional sensitivity. These features make our 3D‐printed cilia sensors well‐suited for a wide range of applications, particularly in challenging and harsh conditions.

## 3 Conclusion

We present an inter‐cilium contact mechanosensing mechanism modeled on artificial conductive cilia. This mechanism uses a graphene‐PCL nanocomposite ink, extrusion‐based 3D‐printing of the ink into high aspect‐ratio cilia arrays, and the responsive electrical properties of these arrays to detect various stimuli. Finite element analysis, along with experimental results, is used to characterize the physical bending of the cilia when linear pressures and external point forces are applied. Reproducibility tests demonstrate high consistency and durability in cilia construction and responsivity. The cilia exhibit excellent resilience, returning to their original unbent position even after being bent 30°, withstanding high flow rates in both air and water, reaching 57 m s^−1^ and 240 mm s^−1^, respectively, demonstrating resistance to aqueous corrosion, and maintaining consistent performance over numerous cyclical loads. Key advantages of this mechanism are its scalability, customizability, and versatility, which set it apart from other mechanosensing cilia sensors based on magnetism or piezoelectricity. The bendable, conductive cilia in this mechanism can exist in two states: contact and separation. As a result, the cilia sensor can detect any stimulus that differentiates between these two states, from changes in surface topology to airflow, water flow, the motion of two surfaces with respect to one another, and beyond. This multifunctionality is both cost‐effective and scalable when compared to specialized pressure or airflow sensors.

In the future, 3D printing could be used to customize patient‐specific designs for this project, which could lead to personalized healthcare and monitoring. Cilia‐based sensors to read Braille dynamically and accurately could be invaluable for the visually impaired or in robotics. Additionally, implantable cilia‐based eyelashes to detect and trigger debris removal could provide effective therapeutics for those with alopecia or in the robotic protection of sensitive camera lenses. Ultimately, this cilia‐based sensing mechanism provides numerous opportunities for 3D‐printed, next‐generation mechanosensing by being applicable to a wide range of disciplines, from environmental studies to the automotive industry, medical rehabilitation, and industrial maintenance.

## 4 Experimental Section

### Ink Preparation

The cilia were printed with various combinations of the polymer PCL, graphene, and the solvent DCM. PCL (Sigma Aldrich) and graphene nanoparticles (Sigma Aldrich) were dissolved in DCM (Sigma Aldrich) for a day. Then the mixture was placed into a planetary centrifugal mixer (AR‐100; Thinky) at 1400 rpm for 240 s, removed and stirred well, and then centrifuged for another 240 s. The electrodes and cilia cups were printed with a two‐part silver microparticle ink (Atom adhesives). The dermal rubber layer was printed with Dragon Skin (Smooth‐On). All devices were printed on a flexible tape substrate (Flex‐Tape).

The optimal ratio of PCL to DCM was found to be 30% PCL by weight (prior to adding graphene). This ratio was used for all cases of pure PCL. Four variations of the ink with increasing concentrations of graphene were synthesized and studied. The inks were denoted PCLG1, PCLG2, PCLG3, and PCLG4 corresponding to weight percentages of graphene ≈3.5%, 6.5%, 8.5%, and 10.5%.

### Material Characterization

A scanning electron microscope (SU‐70 FE‐SEM; Hitachi) at 10 kV was used to obtain micrographs. Tensile and compressive moduli were measured with a texture analyzer (TA. XT plusC; Stable Micro Systems). Young's moduli were calculated from the elastic region during compression. The compression and tension tests were performed with constant parameters, including a constant test speed with an iteratively increasing strain percentage. Tensile grips were used for all tension measurements. A cylindrical 1 cm^2^ stainless steel probe was used for compression measurements.

### Printing Cilia Array Sensors

Cilia sensors were printed with a custom robot gantry (A351, Physik Instrumente L.P.). The Gantry unit has precise control in *X*, *Y*, and *Z* dimensions, has multiple motors in *Z* to allow for multimaterial printing, and operates in printing pressures from 0.1–300 psi using three independent pneumatic dispensing systems (Ultimus V; Nordson EFD; OH, USA). The silver electrodes were printed with a two‐part curable silver epoxy (Atom Adhesives) with a 100 µm stainless steel nozzle with various shapes depending on the application. The cilia were printed vertically in different configurations with various nozzle sizes, including 100, 150, 200, 300, and 1000 µm, all stainless steel, depending on the application with the synthesized PCL/DCM/Graphene ink. After the cilia were printed, a dermal layer of Dragon‐Skin (Smooth‐On) was patterned with a 500 µm stainless steel nozzle at a thickness of roughly 500 µm to fix the cilia in place. When necessary, some cilia were manually given bulbous silver caps (Atom Adhesives).

### Measurements

Electrical conductivity measurements were performed with a source meter (2470 High Voltage SMU; Keithley). Conductivity was calculated from the linear region of a current–voltage sweep which was taken from −2 to 2 V for each sample. Raman spectra were measured by a Raman Spectrometer (LabRAM HR Evolution; Horiba) with a 532 nm laser on graphene‐PCL composites. Contact angle measurements were taken with a goniometer (OCA 15, DataPhysics, USA). XPS spectra were obtained with a scanning XPS Microprobe (VersaProbe III; PHI) on a bead of pure PCL, graphene powder, and dried composites of graphene and PCL with a DCM solvent. All current–time measurements were taken with a bias voltage of 2 V with a sampling rate of 0.01s.

### FEA Simulation

FEA was performed to validate theoretical models of cantilever bending. A cilium of appropriate dimensions (100 µm diameter, 10 mm length) and with the mechanical properties of PCL (Elastic modulus of 363.4 MPa, Ultimate tensile strength 10.5 MPa, Strain at break 0.043)^[^ ^62^ ^]^ was modeled in CAD software (Fusion 360) and subjected to a point force and a linear pressure. The simulation conditions were identical to the experimental parameters, with a linear pressure of 115 mN m^−1^ and a point force of 7.5 mN for the two cases. The displacement of the cantilevers was given by the thermometer and coloring.

The cilia used in Figure 3a–d were specifically designed as artificial constructs without any inclusion of graphene nanofiller. The purpose of this design choice was to initially examine the bending behavior of the cilia in their pure form, free from any influence that the presence of graphene nanopowder might have on their stiffness or the uniformity of FEA simulation results. However, to validate the predicted bending shape of the cilia in the context of actual sensing demonstrations targeted to specific applications, PCL/graphene cilia (Figure 3e) were introduced. These cilia aligned with the proposed framework, serving as a confirmation of the predictions.

## Conflict of Interest

The authors declare no conflict of interest.

## Supporting information

Supporting Information

Supplemental Movie 1

Supplemental Movie 2

Supplemental Movie 3

Supplemental Movie 4

Supplemental Movie 5

Supplemental Movie 6

Supplemental Movie 7

## Data Availability

The data that support the findings of this study are available from the corresponding author upon reasonable request.
