# Supplementary material for: 3D‐Printed Artificial Cilia Arrays: A Versatile Tool for Customizable Mechanosensing
Source: Adv Sci (Weinh). 2023 Jul 23;10(26):2303164. doi: 10.1002/advs.202303164 (PMC10502633; doi:10.1002/advs.202303164)
Supplement: Supplementary file 1 — Supporting Information [file ADVS-10-2303164-s002.pdf]

ADVANCED SCIENCE
Open Access

Supporting Information

for *Adv. Sci.*, DOI 10.1002/advs.202303164

# 3D-Printed Artificial Cilia Arrays: A Versatile Tool for Customizable Mechanosensing

*Phillip Glass, Andy Shar, Charles Pemberton, Ethan Nguyen, Sung Hyun Park and Daeha Joung**

# Supporting Information

**3D-Printed Artificial Cilia Arrays: A Versatile Tool for Customizable Mechanosensing**

*Phillip Glass, Andy Shar, Charles Pemberton, Ethan Nguyen, Sung Hyun Park, and Daeha Joung**

This Supporting Information includes: Printing process for 3D printed cilia sensor; dynamic sensing measurements with a 10×10 cilia arrays; stress-strain curves of pure polycaprolactone (PCL) at different wt% concentrations; current-voltage and stress-strain curves of representative PCL/graphene composites at different graphene concentrations; Raman spectra and X-Ray photospectroscopy (XPS) of PCL/graphene (PCLG) composites at different graphene concentrations; photo images of 10×10 cilia arrays on flexible substrates; water corrosion test of silver epoxy electrodes; force as a function of bending distance for a single cilium of PCL; system of equations for cilia contact as a function of width, height and inter-cilia distance; finite element analysis (FEA) simulations of a single cilium bent with and without a cap; MATLAB code for 3D plot of preceptive sensing; movie of 3D printing a sample cilia sensor; tables for comparison of dynamic and statics sensors with previous ciliary sensors in the literature; movie of cup size demonstrations; movie showing positive effect of silver caps on sensitivity; movie of restoration process after upon bending; movie of 3D printing eyelash; movie of a plant leaf motion sensor; movie of stirring experiments at three different rates.

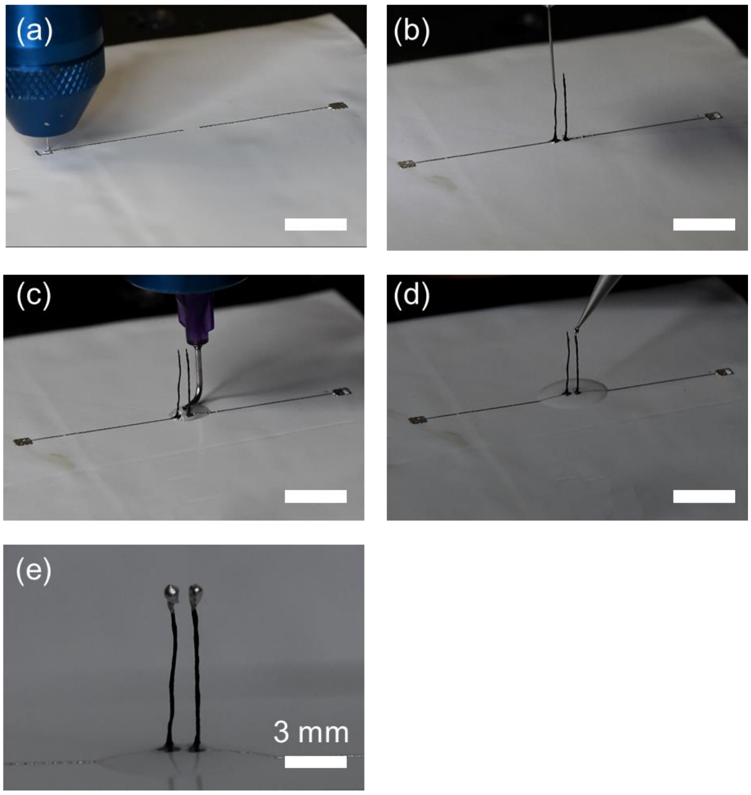


**Figure S1.** The printing process for 3D printed cilia sensor. (a) Silver two-part epoxy prints electrode pads connecting pathways to cups that house the cilia. These cups allow current to flow from the silver through the cilia and still enable the cilia to adhere strongly to the rubberized substrate. (b) Cilia are printed vertically at a high aspect ratio in the cups with a larger bulbous base to promote good contact with the silver and adhesion with the substrate. (c) The dermal layer of flexible silicone (Dragon-Skin$^{TM}$) acts like human skin and holds the cilia firmly in place without disturbing the electrical pathway. (d) 'Caps' are added to some cilia to promote a higher contact area and ensure current flow. (e) A finished 2×1 array of high aspect ratio cilia with caps.

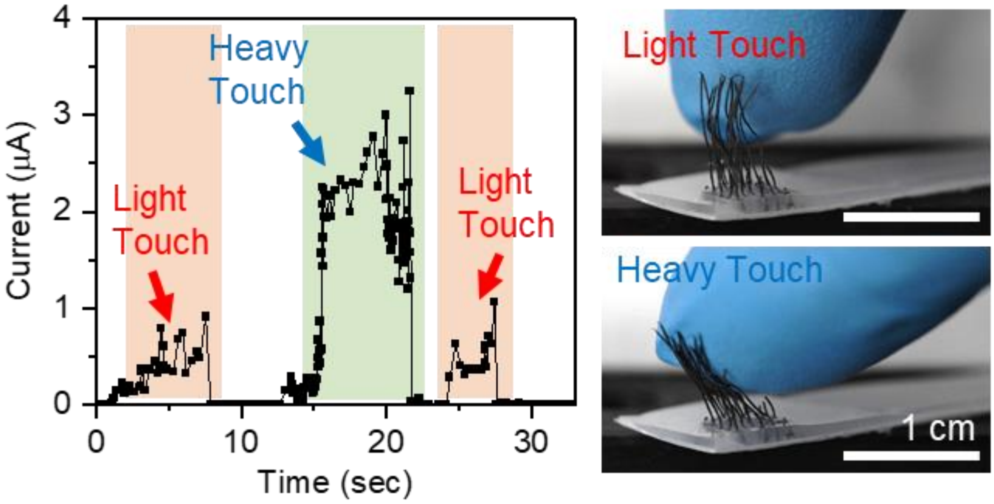


**Figure S2.** Larger arrays of 10×10 cilia can enable receptive sensing applications, which still ensure contact with adjacent cilia without the need for caps sliding past each other in sequence. A current-time graph illustrates the different responses of light and heavy touches, showing a distinct increase in current in the latter.

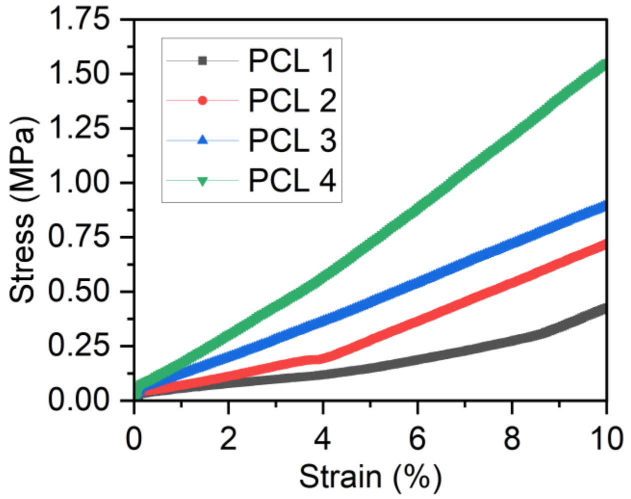


**Figure S3.** Stress-strain curve of pure polycaprolactone (PCL) at increasing concentrations of wt% 10, 20, 30, and 40%, labeled PCL1, PCL2, PCL3, and PCL4, respectively. The four curves correspond to Young's Moduli of 3.88, 7,02, 8.73, and 15.95 MPa, respectively.

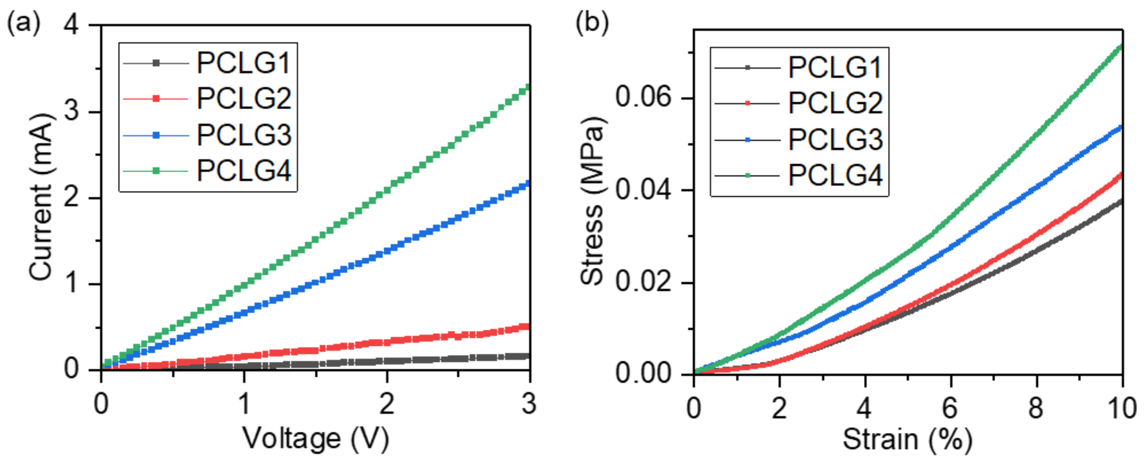


**Figure S4.** (a) Current-voltage (I-V) data for four PCL/graphene (PCLG) composites with graphene wt% of ~ 3.5, 6.5, 8.5, and 10.5%, respectively. Conductivity has a marked increase with graphene concentration, and the I-V relationship is linear (Ohmic) for voltages within 3V. (b) Stress-strain curve for compression of PCLG composites.

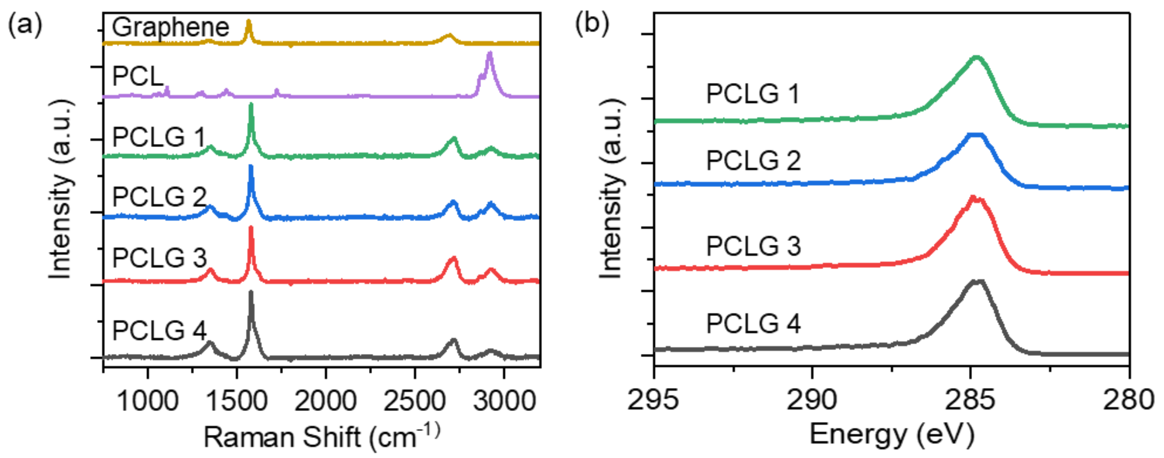


**Figure S5.** (a) Raman spectroscopy of pure graphene, pure PCL, PCLG1, PCLG2, PCLG3, and PCLG4. The characteristic peaks for PCL and graphene are both present in each composite, confirming their chemical character. The four composites are similar in shape and show no significant energetic shift with increasing graphene concentration. (b) X-Ray photospectroscopy (XPS) of the composites near the characteristic carbon peak. There is no significant change in the energetic location of the C-C peak at 284.8 eV.

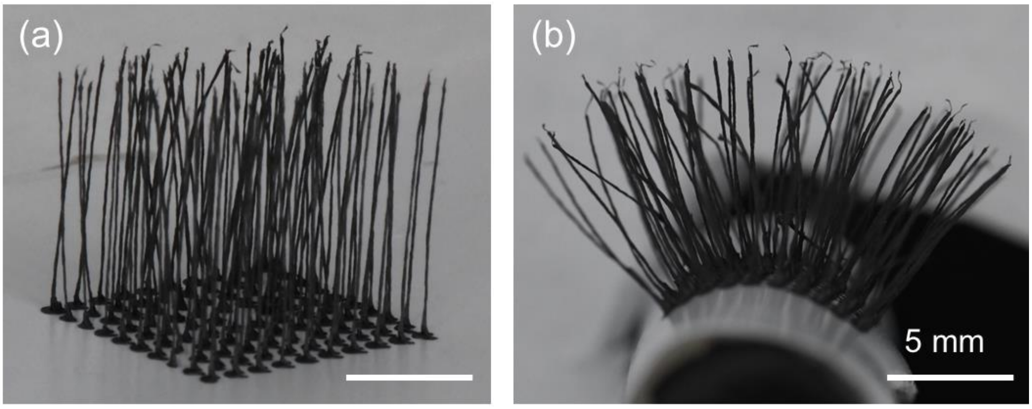


**Figure S6.** Large 10×10 array of cilia (100 µm diameter, 1 cm length). (a) 10×10 of cilia on a rubberized substrate. (b) Cilia bending with the flexible substrate forms a radial orientation.

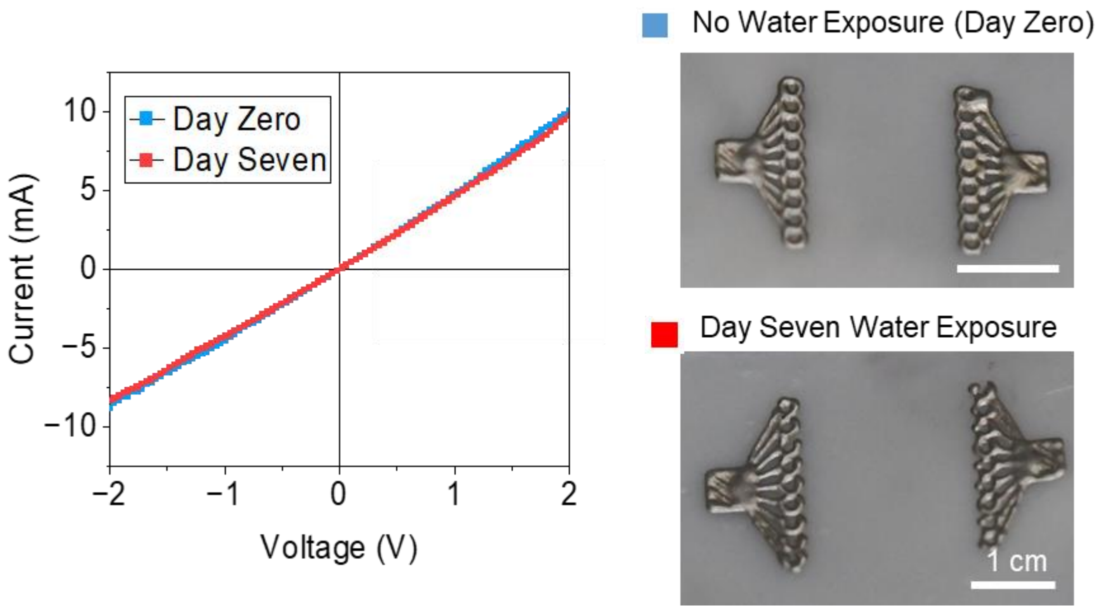


**Figure S7.** Water corrosion test of the silver epoxy electrode. Two sets of electrode patterns were printed with the silver epoxy used in each of the sensors. One set was submerged in deionized (DI) water for a week and removed, while another was left in air. The I-V curves are nearly identical, indicating no corrosion by water exposure to the sensor's metal element.

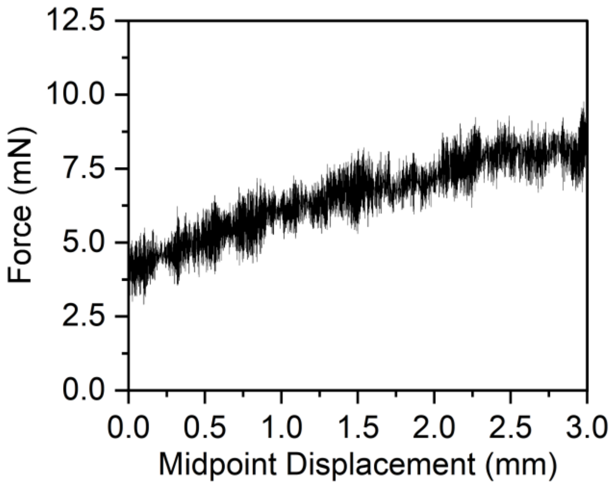


**Figure S8.** Applied forces as a function of bending distance for a single cilium of PCL. This experimentally provides the minimum force required to bend one cilium 1 mm to the nearest unbent cilium. This data is used to inform simulation in FEA.

$$L = \frac{H(2D+W)}{2(D-W)}, H = \frac{2L(D-W)}{(2D+W)}, D = \frac{W(2L+H)}{2(L-H)}, W = \frac{2D(L-H)}{(2L+H)}$$

**Equation S1.** System of equations parameterizing the minimum bending required to facilitate contact between a cilium of length *L* and an unbent cilium *D* distance away when bent by a width *W* and height *H* feature.

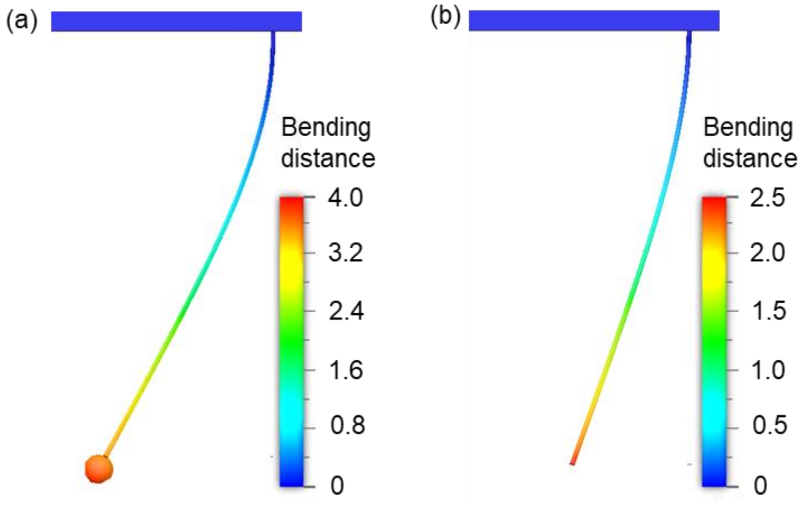


**Figure S9.** Finite element analysis (FEA) of two cilia bent under 1 mN of force with and without a cap. The cilia were simulated using the same physical parameters as in Figure 2 (details in the experimental section). They were subjected to 1 mN of force at their tip, with the substrate they are adhered to fixed from moving. The cilia are 100 µm in diameter, and the cap is 300 µm in diameter. (a) The capped cilium has a higher moment of inertia and bends more at its tip (~ 4 mm) in response to the same force than the (b) uncapped cilium does (~ 2.5 mm).

```
x = import_Data{:,1}; % reads 1st column data
y = import_Data{:,2}; % reads 2nd column data
z = import_Data{:,3}; % reads 3rd column data

% get vector of data
xlin = linspace(min(x), max(x), 50);
ylin = linspace(min(y), max(y), 50);

% get grid coordinates from xlin and ylin
[X,Y] = meshgrid(xlin, ylin);

Z = griddata(x,y,z,X,Y, 'nearest'); % can change 'nearest' to other values

mesh(X,Y,Z)
```

**Figure S10.** MATLAB code which inputs Excel data for X, Y, and Z coordinate systems and creates a corresponding mesh plot.

**Table S1.** Comparison of cilia-based scanning sensors (Dynamic sensors).

| Sensor Structure | Fabrication Requirements | Max Force Applied | Sensitivity | Young's Modulus | Dimensions | [Ref] |
|---|---|---|---|---|---|---|
| Metallic needles embedded in double-layered magnetic PDMS | 90°C for >1h vacuum treatment | 60 µN | 630 uT/mN | 0.5-1.2 MPa | *d*=120 µm, *l*=4.5 mm | [S1] |
| Iron NW-PDMS cilia on a GMR sensor | Apparatus for electro & ion beam deposition, ion milling | 55 mN | 0.8 mV/mN | 0.76 MPa | *d*=200 µm, *l*=1 mm | [S2] |
| NdFeB-PDMS cilia on a GMR sensor | Apparatus for electro & ion beam deposition, ion milling | 28.5 mN | 0.26 mV/mN | 1 MPa | *d*=200 µm, *l*=1 mm | [S3] |
| Graphene-PCL cilia arrays on variable substrates | Room temperature 3D printing, <15 minutes total, planetary mixer | 27 mN | 57 µA/mN | 0.7 MPa | *d*=100-1,000 µm, *l*=1-20 mm | This Work |

* PDMS: polydimethylsiloxane, NW: nanowire, GMR: giant magneto-resistive, NdFeB: neodymium magnet, PCL: polycaprolactone, *d*: diameter, *l*: length (height)

**Table S2.** Comparison of cilia-based airflow sensors (Static sensors).

| Sensor Structure | Fabrication Requirements | Max Air Velocity | Sensitivity | Young's Modulus | Dimensions | Direction Sensitive | [Ref] |
|---|---|---|---|---|---|---|---|
| Metallic needles embedded in double-layered magnetic PDMS | 90°C for >1h Vacuum conditions | 30 m/s | 630 uT/mN | 0.5-1.2 MPa | *d*=120 µm, *l*=4.5 mm | No | [S1] |
| PDMS cantilever structure with GNP piezoresistive element | 3D printing, vacuum desiccation, 48hr cure, annealing at 120°C | 10 m/s | 5800 Ω/(m/s) | Not measured | *d*=400 µm, *l*=8 mm | No | [S4] |
| Polyurethane and PDMS cilia arrays on GNP sensor films | Reactive ion etching, thermal baking and curing at up to 120° C, vacuum desiccation | 200 kPa | $10^{-4}$ $(\Delta I/I_0)$/kPa | 2, 19.8 MPa | *d*=500 µm, *th*=300 *l*=10 mm | No | [S5] |
| Graphene-PCL cilia arrays on variable substrates | Room temperature 3D printing, <15 minutes total, planetary mixer | 57 m/s | $10^3$ $(\Delta I/I_0)$ $/m\cdot s^{-1}$ | 0.7 MPa | *d*=100-1,000 µm, *l*=1-20 mm | Yes | This Work |

* PDMS: polydimethylsiloxane, GNP: graphene nanoplatelets, PCL: polycaprolactone, *d*: diameter, *l*: length (height), *th*: Thickness

**Table S3.** Comparison of cilia-based water flow sensors (Static sensors).

| Sensor Structure | Fabrication Requirements | Max Water Velocity | Sensitivity | Young's Modulus | Dimensions | [Ref] |
|---|---|---|---|---|---|---|
| PDMS cantilever structure with GNP piezoresistive element | 3D printing, vacuum desiccation, 48hr cure, annealing at 120°C | 90 mm/s | 30 mV/(m·s$^{-1}$) | Not measured | *d*=400 µm, *l*=8 mm | [S4] |
| Titanium pillar rooted in circular CNF membrane | Electrospinning apparatus, 750°C under nitrogen for pyrolization | 66 mm/s | 26 mV/(m·s$^{-1}$) | Not measured | Not stated | [S6] |
| Vertically aligned CNT bundles positioned on PDVF nanofiber sensing membrane | Incubation at 70°C for >1hr, electrospinning apparatus, overnight stirring | 100 mm/s | 45 mV/(m·s$^{-1}$) | 2.2 GPa | *d*=350 µm, *l*=4 mm | [S7] |
| Graphene-PCL cilia arrays on variable substrates | Room temperature 3D printing, <15 minutes total, planetary mixer | 240 mm/s | 29 µA/(m·s$^{-1}$) | 0.7 MPa | *d*=100-1,000 µm, *l*=1-20 mm | This Work |

* PDMS: polydimethylsiloxane, GNP: graphene nanoplatelets, CNF: electrospun carbon nanofiber, CNT: carbon nanotube, PDVF: polyvinylidene fluoride, PCL: polycaprolactone, *d*: diameter, *l*: length (height)

**References**

[S1] J. Man, J. Zhang, G. Chen, N. Xue, J. Chen, *Microsyst. Nanoeng.* **2023**, *9*, 1.
[S2] A. Alfadhel, M. A. Khan, S. Cardoso de Freitas, J. Kosel, *IEEE Sens. J.* **2016**, *16*, 8700.
[S3] P. Ribeiro, M. A. Khan, A. Alfadhel, J. Kosel, F. Franco, S. Cardoso, A. Bernardino, A. Schmitz, J. Santos-Victor, L. Jamone, *IEEE Robot. Autom. Lett.* **2017**, *2*, 971.
[S4] A. M. Kamat, X. Zheng, B. Jayawardhana, A. G. P. Kottapalli, *Nanotechnology* **2020**, *32*, 095501.
[S5] S. Chun, W. Son, C. Choi, H. Min, J. Kim, H. J. Lee, D. Kim, C. Kim, J. Koh, C. Pang, *ACS Appl. Mater. Interfaces* **2019**, *11*, 13608.
[S6] D. Sengupta, D. Trap, A. G. P. Kottapalli, *Nanomaterials* **2020**, *10*, 211.
[S7] M. Bora, A. G. P. Kottapalli, J. Miao, M. S. Triantafyllou, *NPG Asia Mater.* **2017**, *9*, e440.
